# Protective Effect of Val_129_-PrP against Bovine Spongiform Encephalopathy but not Variant Creutzfeldt-Jakob Disease

**DOI:** 10.3201/eid2309.161948

**Published:** 2017-09

**Authors:** Natalia Fernández-Borges, Juan Carlos Espinosa, Alba Marín-Moreno, Patricia Aguilar-Calvo, Emmanuel A. Asante, Tetsuyuki Kitamoto, Shirou Mohri, Olivier Andréoletti, Juan María Torres

**Affiliations:** Centro de Investigación en Sanidad Animal, Instituto Nacional de Investigación y Tecnología Agraria y Alimentaria (CISA-INIA);; Valdeolmos, Madrid, Spain (N. Fernández-Borges, J.C. Espinosa, A. Marín-Moreno, P. Aguilar-Calvo, J.M. Torres);; MRC Prion Unit, Department of Neurodegenerative Disease, University College London, Institute of Neurology, London, UK (E.A. Asante);; Tohoku University Graduate School of Medicine, Sendai, Japan (T. Kitamoto, S. Mohri);; UMR INRA ENVT 1225, Interactions Hôtes Agents Pathogènes, Ecole Nationale Vétérinaire de Toulouse, Toulouse, France (O. Andréoletti)

**Keywords:** Bovine spongiform encephalopathy, BSE, prion susceptibility, PrP, Val129-PrP, transmission barrier, variant Creutzfeldt-Jakob disease, vCJD, zoonoses, PRNP, methionine, valine, Met/Val, zoonoses, transmissible spongiform encephalopathies, ruminant, prions

## Abstract

Bovine spongiform encephalopathy (BSE) is the only known zoonotic prion that causes variant Creutzfeldt-Jakob disease (vCJD) in humans. The major risk determinant for this disease is the polymorphic codon 129 of the human prion protein (Hu-PrP), where either methionine (Met_129_) or valine (Val_129_) can be encoded. To date, all clinical and neuropathologically confirmed vCJD cases have been Met_129_ homozygous, with the exception of 1 recently reported Met/Val heterozygous case. Here, we found that transgenic mice homozygous for Val_129_ Hu-PrP show severely restricted propagation of the BSE prion strain, but this constraint can be partially overcome by adaptation of the BSE agent to the Met_129_ Hu-PrP. In addition, the transmission of vCJD to transgenic mice homozygous for Val_129_ Hu-PrP resulted in a prion with distinct strain features. These observations may indicate increased risk for vCJD secondary transmission in Val_129_ Hu-PrP–positive humans with the emergence of new strain features.

The presence of variant Creutzfeldt-Jakob disease (vCJD) is considered by strong epidemiologic, pathologic, and molecular evidence to be a likely consequence of human dietary exposure to the bovine spongiform encephalopathy (BSE) agent (*1*–*3*). Secondary vCJD infection has occurred through iatrogenic routes such as blood transfusion (*4*–*7*). The pathogenesis of these fatal transmissible spongiform encephalopathies (TSEs), called prion diseases, is associated with the accumulation of the abnormal isoform (PrP^Sc^) of prion protein (PrP), which is converted from the normal cellular isoform (PrP^C^) (*8*). This conversion process involves a posttranslational conformational change of PrP^C^ and PrP^Sc^ that can be distinguished biochemically from PrP^C^ by its partial resistance to proteolysis and detergent insolubility (*9*,*10*).

The neuropathological features of vCJD are characterized by the presence of abundant florid PrP plaques and the propagation of type 4 disease-related PrP^Sc^ in the brain (*1*,*11*). Differences in the level of glycosylation, as well as in the size of protease-digested PrP^Sc^, are widely used as surrogates of prion strain typing; 2 main classifications are recognized in the prion field (*1*,*12*). According to 1 of these classifications (*1*,*13*), type 4 PrP^Sc^ is characterized by a fragment size and glycoform ratio similar to that seen in BSE and BSE transmitted to several other species, with a predominance of the diglycosylated PrP glycoform (*1*,*13*–*15*).

Polymorphism at codon 129 of the human PrP gene (*PRNP*), where methionine (Met) or valine (Val) can be encoded, strongly affects susceptibility to human prion diseases (*16*–*20*). vCJD has only been neuropathologically confirmed in persons homozygous for Met at residue 129 of human PrP (*21*), with 1 exception of heterozygosity (Met/Val) at this codon (*22*). In addition, asymptomatic peripheral involvement in vCJD infection has been reported in 2 Met/Val_129_–positive persons (*5*,*7*). Retrospective studies of the prevalence of subclinical vCJD infection using appendectomy and tonsillectomy specimens in the United Kingdom described 6 appendixes that were positive for disease-associated prion protein in Val/Val_129_ persons (*23*–*25*). All of these human studies, in addition to the extremely prolonged and variable incubation periods seen in prion transmission experiments when crossing a species barrier, suggest that persons encoding any of the 3 human PrP codon 129 genotypes may be susceptible to vCJD, including secondary vCJD transmitted through blood transfusion, blood products, tissue and organ transplantation, and other iatrogenic routes.

Because only 1 definite case of heterozygous Met/Val_129_ vCJD and no homozygous Val_129_ cases have been described, it is unknown whether the clinicopathologic characteristics and biochemical properties of vCJD would appear in persons with these codon 129 genotypes. To gain insights into that question, vCJD/BSE transmission studies in which either humanized overexpressing or knock-in transgenic mice were used have been performed (*2*,*26*–*30*). However, some discrepancies in the transmission efficiency of vCJD to humanized knock-in transgenic mice can be found, depending on the origin of the mice and on the vCJD isolate (*29*,*30*). Previous studies in humanized overexpressing transgenic mice revealed that the 3 human PrP codon 129 genotypes can be infected with vCJD but show significant differences depending on the genotype. Moreover, mice with the Val/Val_129_ genotype were more susceptible to vCJD infection than expected but lack the neuropathological characteristics observed with Met/Met_129_ (*2*,*26*–*28*).

In this study, we evaluated the zoonotic potential of BSE and BSE adapted to different species by using transgenic mice overexpressing similar levels of human PrP^C^ carrying Met/Met, Met/Val, or Val/Val at position 129 of human PrP. Furthermore, we used these models to re-evaluate the potential for human-to-human spread of vCJD, as well as the differential susceptibility and characteristics of the transmitted disease across the different *PRNP* codon 129 genotypes in humans.

## Materials and Methods

### Ethics Statement

We carried out animal experiments in strict accordance with the recommendations in the guidelines of the Code for Methods and Welfare Considerations in Behavioral Research with Animals (Directive 86/609EC and 2010/63/EU), and all efforts were made to minimize suffering. Experiments were approved by the Committee on the Ethics of Animal Experiments of the Instituto Nacional de Investigación y Tecnología Agraria y Alimentaria (Madrid, Spain; permit nos. CEEA2012/024 and CEEA2009/004).

### TSE Isolates

We used 11 isolates from different sources in this study (Table 1 [*31**–**39*]). For mouse inoculation, we prepared all isolates from brain tissues as 10% weight/volume (wt/vol) homogenates in 5% glucose. We performed second passages by inoculating transgenic mice with 10% (wt/vol) homogenates in 5% glucose of brains selected from passage 1.

**Table 1:** Description of prion isolates used in analysis of bovine spongiform encephalopathy and Creutzfeldt-Jakob disease*

Isolate	Sample codification	Description (reference)	Supplier†
Hu-sCJD MM1	BC 1011	sCJD PrP-Met_129_ type 1 human natural case	BHUFA
Hu-sCJD VV2	BC 1452	sCJD PrP-Val_129_ type 2 human natural case	BHUFA
Hu-TSE negative		TSE free human brain	NIBSC
Ca-BSE_0_	Fr (139)	BSE naturally infected cow (*31*,*32*)	INRA
Ca-BSE_2_	UK (PG1199/00)	BSE naturally infected cow (*33*,*34*)	VLA
Ca-BSE_0_/TgPo	Ca-BSE_0_/Tg001	BSE transmitted experimentally to porcine transgenic mice (*32*)	CISA
Ca-BSE_0_/Sh(ARQ)	Fr (ARQ0)	Pool of brains from terminally ill ARQ/ARQ sheep inoculated with Ca-BSE (*31*,*32*)	INRA
Ca-BSE/Go	gBSE-P12	Pool of brains from 3 terminally ill wild type goats inoculated with a mixture of 4 cattle-BSE field cases (*35*–*37*)	Roslin
Go-BSE	Fr (CH636)	Goat BSE case (*38*)	AFSSA
Hu-vCJD_1_	UK (NHBY/0014)	vCJD PrP-Met_129_ human infected case (*39*)	NIBSC
Hu-vCJD_2_	BC 1458	vCJD PrP-Met_129_ human infected case	BHUFA

*BSE, bovine spongiform encephalopathy; Ca, cattle; CJD, Creutzfeldt-Jakob disease; Go, goat; Hu, human; Met_129_, methionine; PrP, prion protein; sCJD, sporadic CJD; TSE, transmissible spongiform encephalopathy; Val_129_, valine; vCJD, variant CJD. †AFSSA, Agence Française de Sécurité Sanitaire des Aliments National TSE Reference Laboratory, Lyon, France; BHUFA, Biobanco Hospital Universitario Fundación Alcorcón, Madrid, Spain; CISA, Centro de Investigación en Sanidad Animal, Madrid, Spain; INRA, French National Institute for Agricultural Research, Nouzilly, France; NIBSC, National Institute for Biologic Standards and Control Creutzfeld-Jakob Disease Resource Centre, South Mimms, Potters Bar, United Kingdom; Roslin, The Roslin Institute and Royal (Dick) School of Veterinary Studies, University of Edinburgh, Easter Bush, Midlothian, United Kingdom; VLA, Veterinary Laboratory Agency, New Haw. Addlestone, Surrey, United Kingdom.

### Mouse Transmission Studies

We inoculated all isolates in 3 different transgenic mouse models: 1) HuPrP-Tg340-Met_129_ (TgMet_129_) mouse line expressing human Met_129_-PrP^C^ variant (*31*); 2) HuPrP-Tg361-Val_129_ (TgVal_129_) mouse line expressing human Val_129_-PrP^C^ variant (*40*); and 3) HuPrP-Tg351-Met/Val_129_ (TgMet/Val_129_) transgenic mouse line obtained by mating TgMet_129_ and TgVal_129_ mice (*40*). All of these transgenic lines show similar brain expression levels of PrP^C^ (around 4-fold the level of expression in the human brain) on a mouse PrP null background. We performed additional inoculations in HuPrP-Tg362-Val_129_, a transgenic mouse line expressing 8-fold the level of PrP^C^ expression in human brain (TgVal_129_ [8×]) (*41*). We performed subsequent bioassays for the detection of low-level propagation of infectious BSE and BSE-derived prions in BoPrP-Tg110 mice, which are highly susceptible to vCJD prions (*42*,*43*), probably caused by the trace-back phenomenon (*30*).

We anesthetized individually identified mice, 6–7 weeks of age, with isoflurane and inoculated them with a 2-mg equivalent of brain homogenate in the right parietal lobe by using a 25-gauge disposable hypodermic needle. We observed mice daily and assessed neurologic status 2 times per week. When progression of a TSE disease was evident or at the established experimental endpoint (700 days postinoculation [dpi]), we euthanized the animal for ethical reasons and performed necropsy, excising the brain. We then fixed part of the brain by immersion in neutral-buffered 10% formalin (4% 2-formaldehyde) and used the tissue for quantifying spongiform degeneration by histopathology. We froze the remaining tissue at −20°C and used it to determine the presence of disease-associated proteinase K (PK)–resistant PrP (PrP^res^) by Western blot. 

In all cases, we calculated mouse survival time and disease attack rate for each isolate. We expressed survival times as mean ±SD of the dpi for all mice positive for PrP^res^. We defined the attack rate as the proportion of all inoculated mice whose samples tested positive for PrP^res^. We used brain homogenates from PrP^res^–positive mice, where available, for further passaging. When all mice were scored negative for PrP^res^ on primary passage, we used PrP^res^-negative brain homogenates for second passage.

### Western Blot

We homogenized frozen brain tissues (175 ± 20 mg) in 5% glucose in distilled water in grinding tubes (Bio-Rad, Hercules, CA, USA) adjusted to 10% (wt/vol) by using a TeSeE Precess 48TM homogenizer (Bio-Rad), according to the manufacturer’s instructions. We determined presence of PrP^res^ in transgenic mouse brains by Western blot, using the reagents of the ELISA commercial test TeSeE (Bio-Rad). Based on a previously described protocol (*31*), we treated 10–100 μL of 10% wt/vol brain homogenates with proteinase K; the resulting samples were loaded in 12% Bis-Tris Gel (Criterion XT; Bio-Rad). We transferred proteins electrophoretically onto PVDF membranes (Millipore, Billerica, MA, USA), which were blocked overnight with 2% BSA blocking buffer (Sigma-Aldrich, St. Louis, MO, USA). For immunoblotting, we incubated with Sha 31 (*44*) monoclonal antibody (mAb) at a concentration of 1 µg/mL to identify the 145-WEDRYYRE-152 epitope of the human PrP^C^ sequence. To detect immunocomplexes, we incubated the membranes for 1 h with horseradish peroxidase conjugated anti-mouse IgG (GE Healthcare Amersham Biosciences, Little Chalfont, UK). Immunoblots were developed with enhanced chemiluminiscence ECL Select (GE Healthcare Amersham Biosciences). Images were captured using the ChemiDoc WRS+ System (Bio-Rad) and processed using Image Lab 5.2.1 software (Bio-Rad).

### Histopathological Analysis

We performed procedures for the histopathological analysis of mouse brains as previously described (*45*). We immediately fixed mouse brain samples in neutral-buffered 10% formalin (4% 2-formaldehyde) during necropsy and embedded the tissues in paraffin later. After deparaffinization, we stained 2 μm–thick tissue slices with hematoxylin and eosin and established lesion profiles by using published standard methods (*46*). We conducted paraffin-embedded tissue (PET) blots as previously described (*47*).

## Results

### BSE Resistance in TgVal_129_ Mice 

To evaluate the relative susceptibility of the 3 human *PRNP* codon 129 genotypes to BSE, we performed serial transmission studies in 3 transgenic mouse lines expressing human PrP. These mouse lines were homozygous for Met (TgMet_129_) or Val (TgVal_129_) at codon 129 of human PrP or were their F1 cross (TgMet/Val_129_). These mouse models expressed similar human PrP levels, ≈4-fold more than that seen in uninfected human brain tissue (*40*). We observed no clinical signs of prion disease or PrP^res^ accumulation in control mice inoculated with TSE-free control brain homogenate. The 3 human transgenic mouse models were readily infected when inoculated with sporadic CJD (sCJD) (Table 2). The 2 sCJD cases used as inocula in this study were classified as Met_129_ type 1 (Hu-sCJD MM1) and Val_129_ type 2 (Hu-sCJD VV2) (*12*) on the basis of the patient´s *PRNP* genotype at codon 129 and the PrP^res^ Western blot profiles of these samples.

**Table 2:** Transmission of cattle, porcine, sheep, and goat BSE isolates to mice in transgenic mouse lines TgMet129, TgMet/Val129, and TgVal129*

Isolates	Mean survival time, d ±SD (no. PrP^res^-positive/inoculated animals) [reference]†‡							
TgMet_129_			TgMet/Val_129_			TgVal_129_	
First passage	Second passage		First passage	Second passage		First passage	Second passage
Hu-sCJD MM1	219 ±17 (6/6) [*40*]	239 ±8 (6/6) [*40*]		243 ±14 (6/6) [*40*]	260 ±13 (6/6) [*40*]		327 ±19 (6/6) [*40*]	286 ±16 (6/6) [*40*]
Hu-sCJD VV2	618 ±81 (6/6) [*40*]	509 ±41 (6/6) [*40*]		588 ±74 (6/6) [*40*]	594 ±86 (6/6) [*40*]		168 ±12 (6/6) [*40*]	169 ±12 (6/6) [*40*]
Hu-TSE negative	>700‡ (0/6)	>700‡ (0/6)		>700‡ (0/6)	>700‡ (0/6)		>700‡ (0/6)	>700‡ (0/6)
Ca-BSE_0_	739 (1/6) (*31*)	633 ±32 (6/6)		>700‡ (0/6)	>700‡ (0/6)		>700‡ (0/6)	>700‡ (0/6)
Ca-BSE_2_	491–707 (0/9) [*31*]	572 ±37 (3/4) [*31*]		>700‡ (0/5)	ND		>700‡ (0/3)§	>700‡ (0/3)§
Ca-BSE/TgPo	653 ±45 (3/5)	ND		ND	ND		>700‡ (0/6)	ND
Ca-BSE/Sh(ARQ)	615 ±84 (4/6) [*31*]	564 ±39 (5/5) [*31*]		>700b (0/6)	>700‡ (0/6)		>700‡ (0/6)	>700‡ (0/6)
Ca-BSE/Go	>700‡ (5/5)	>700‡ (5/5)		476 (1/10)	ND		>700‡ (0/5)¶	ND
Go-BSE	683 ± 36 (6/6)	675 ± 36 (5/5)		>700‡ (0/6)	ND		>700‡ (0/6)	ND

*BSE, bovine spongiform encephalopathy; Ca, cattle; CJD, Creutzfeldt-Jakob disease; Go, goat; Hu, human; Met_129_, methionine; ND, not done; PrP^res^, proteinase K–resistant PrP; sCJD, sporadic CJD; Val_129_, valine; vCJD, variant CJD.  †Survival time is indicated for all mice that scored positive for PrP^res^ ‡Animals without clinical signs were euthanized at 700 dpi. §Three additional animals were culled before the end of the experiment because of intercurrent illnesses; all were negative for brain PrP^res^ by using Western blot. ¶Positive subclinical infection tested in Bo-Tg110 mice.

We inoculated the 3 mouse models intracerebrally with a panel of BSE isolates from different species (cattle, pig, sheep, and goat; Table 2). As previously described in TgMet_129_ mice (*31*), we found a higher transmission efficiency adjudged by comparatively higher attack rates for BSE isolates previously passaged in other species than for cattle BSE, suggesting a strong transmission barrier to cattle BSE in these mice.

At completion of the first and second passages, none of the TgVal_129_ mice challenged with the different BSE isolates developed clinical disease, and no PrP^res^ accumulation was found in their brains (Table 2). Because of intercurrent illnesses, the group of TgVal_129_ mice challenged with Ca-BSE_2_ was considerably reduced in size; however, the absence of transmission to TgVal_129_ mice challenged with a second BSE inocula, Ca-BSE_0_, reinforces this negative result. In addition, results of subsequent passage of brain homogenates from these mice to BoPrP-Tg110 mice were negative, ruling out the presence of subclinical infection, with the exception of TgVal_129_ mice inoculated with Go-BSE. For this isolate, 3 of 6 BoPrP-Tg110 mouse brains showed detectable PrP^res^ and had a long incubation time of 427 ± 38 dpi, suggesting very low infectivity (Technical Appendix Table 1).

To confirm that the lack of susceptibility of TgVal_129_ mice to cattle BSE and to BSE previously adapted in different species was not caused by inadequate PrP substrate, we used the TgVal_129_ (8×) mouse line (*41*). However, even under these high human PrP expression level conditions, none of the inoculated TgVal_129_ (8×) mice showed any evidence of infection after challenge with the different BSE isolates (Technical Appendix Table 2). This result indicates that even an increase in the TgVal_129_ PrP expression level is not enough to allow transmission of BSE prions, irrespective of the species in which BSE has been previously passaged.

In a similar manner to that seen in the TgVal_129_ mice, we observed no clinical disease and no disease-associated PK-resistant PrP accumulation on first or second passage of the different BSE isolates in TgMet/Val_129_ mice. However, we did observe an exception in 1 TgMet/Val_129_ mouse inoculated with Go-BSE without clinical signs but with a positive score for brain PrP^res^ that died at 476 dpi (Table 2). These findings support the interpretation that human-PrP Val_129_ polymorphism severely restricts propagation of the BSE prion strain independently of the species in which it had previously been adapted.

### BSE Adaptation to the Human PrP Sequence

In parallel to the transmission experiments with the different BSE isolates, we also inoculated the 3 humanized transgenic mouse models with human brain material from 2 different cases of vCJD PrP-Met_129_ (Hu-vCJD_1_ and Hu-vCJD_2_). On first passage, 100% of the TgMet_129_ mice developed clinical disease in response to all inocula in the panel (Table 3). However, only the inoculum Hu-vCJD_2_ previously passaged in TgMet/Val_129_ mice caused clinical disease in the same heterozygous genotype upon serial passages; the rest of the inocula caused only subclinical infections in this genotype (Table 3). 

**Table 3:** Intracerebral inoculation of transgenic mice that express human PrP with vCJD and with vCJD previously adapted in TgMet129 or TgMet/Val129 mice*

Isolates	Mean survival time, d ±SD (no. PrP^res^-positive/inoculated animals) [reference]†		
TgMet_129_	TgMet/Val_129_	TgVal_129_
Hu-vCJD_1_	626 ±29 (6/6) [*31*]	>700‡ (3/3)§	>700‡ (0/5)
Hu-vCJD_1_→TgMet_129_	650 ±60 (4/4)	>700‡ (5/5)	>700‡ (5/5)
Hu-vCJD_2_	545 ±146 (5/5)	>700‡ (5/5)	>700‡ (0/6)#
Hu-vCJD_2_→TgMet_129_	564 ±39 (4/4)	>700‡ (5/5)	>700‡ (2/2)¶
Hu-vCJD_2_→TgMet/Val_129_	601 ±32 (5/5)	651 ±17 (7/7)	>700‡ (7/7)
Ca-BSE_2_→TgMet_129_	614 ±87 (6/6)	>700‡ (4/4)	>700‡ (3/4)
Ca-BSE/Sh(ARQ)→TgMet_129_	534 ±55 (5/6)	>700‡ (5/6)	>700‡ (5/6)
Ca-BSE/Go→TgMet_129_	609 ±67 (5/5)	>700‡ (4/4)	>700‡ (6/6)

*BSE, bovine spongiform encephalopathy; Ca, cattle; CJD, Creutzfeldt-Jakob disease; Go, goat; Hu, human; Met_129_, methionine; PrP, prion protein; PrP^res^, proteinase K–resistant PrP; sCJD, sporadic CJD; Val_129_, valine; vCJD, variant CJD. †Survival time is indicated for all mice scored positive for PrP^res^. ‡Animals were euthanized without clinical signs at 700 dpi. §Three additional animals had to be culled before the end of the experiment because of intercurrent illnesses; all were negative for brain PrP^res^ on WB. ¶Four additional animals had to be culled before the end of the experiment because of intercurrent illnesses; all were negative for PrP^res^ expression on Western blot. #Positive subclinical infection tested in Bo-Tg110 mice.

**Figure 1:**
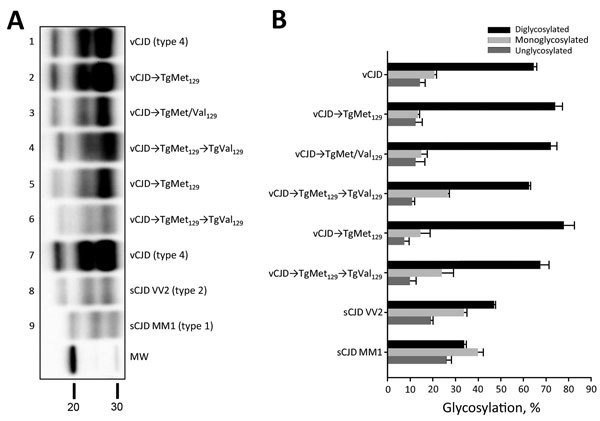
Biochemical features of the protease-resistant prion protein (PrPres) detected in the brain of TgMet129, TgMet/Val129, and TgVal129 mice inoculated with vCJD. A) PrPres detected in TgMet129 (lanes 2 and 5), TgMet/Val129 (lane 3), and TgVal129 (lanes 4 and 6) mice inoculated with vCJD brain homogenate or TgMet129-passaged vCJD prions. Similar quantities of PrPres were loaded for adequate comparison and immunoblots were detected with Sha31 monoclonal antibody (mAb). The original vCJD isolate (Hu-vCJD2) used for mouse inoculations was also included in the blot (lanes 1 and 7). sCJD VV2 and MM1 isolates were included for biochemical comparative purposes (lanes 8 and 9, respectively). Molecular weight (MW) in kDa is shown. B) Glycoform analysis of PrPres from TgMet129, TgMet/Val129 and TgVal129 mice inoculated with vCJD brain homogenate or TgMet129-passaged vCJD prions. PrPres was detected by Western blot testing using the Sha31 mAb, as for panel A. The data shown are the means of >4 measurements in >2 different Western blots using the Image Lab (Bio-Rad, Hercules, CA, USA) program after capture with ChemiDoc XRS+ (Bio-Rad) under nonsaturating conditions. Error bars indicate SD. CJD, Creutzfeldt-Jakob disease; sCJD, sporadic CJD; vCJD, variant CJD.

**Figure 2:**
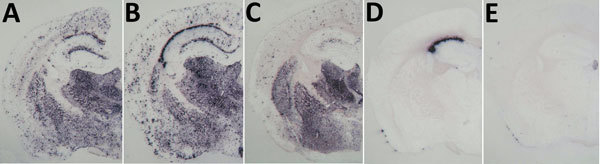
Protease-resistant prion protein distribution pattern in brains of prion protein humanized transgenic mice inoculated with variant Creutzfeldt-Jakob disease (vCJD) on second passage. A, B) TgMet129 mice inoculated with vCJD. C) TgMet/Val129 mice inoculated with vCJD. D, E) TgVal129 mice inoculated with vCJD propagated in TgMet129 mice. Original magnification ×20 for all panels.

The PrP^res^ molecular profile (Figure 1, panel A, lanes 2, 3, and 5; Figure 1, panel B) and the PrP^res^ distribution patterns on paraffin-embedded tissue (PET) blots in the mouse brains (Figure 2, panels A, B, C) were similar in both the TgMet_129_ and TgMet/Val_129_ mice, with or without clinical disease. However, we consistently observed a lower PrP^res^ accumulation in TgMet/Val_129_ mice compared with TgMet homozygous animals, particularly in the hippocampus area, probably caused by a slower conversion rate of PrP^Sc^ in these animals with a half dose of PrP-Met_129_.

In sharp contrast, none of the TgVal_129_ mice challenged with the 2 vCJD primary inocula, Hu-vCJD_1_ and Hu-vCJD_2_, developed clinical disease and no PrP^res^ accumulation was found in their brains after Western blot (WB) analysis (Table 3). However, subsequent passage of brain homogenates from TgVal_129_ mice inoculated with Hu-vCJD_2_ (that remained apparently uninfected) to BoPrP-Tg110 mice showed evidence of subclinical infection. These subpassages led to a mean incubation time of 371 ± 5 dpi and to propagation of PrP^res^ that was detectable by WB in 100% of animals (Technical Appendix Table 1), showing a biochemical pattern indistinguishable from that of cattle BSE infection in this mouse model.

These results suggest that the adaptation of the BSE agent to human PrP sequence could favor its transmission to the polymorphic human PrP Val_129_ genotype. In this context, we passaged all isolates in TgMet_129_ mice before subsequent inoculation in TgVal_129_ mice. Although we did not observe clinical prion disease, the inoculated TgVal_129_ mice had an infection rate remarkably close to 100%, as assessed by the presence of brain PrP^res^ at the end of the experiment (Table 3). We obtained similar results with the Hu-vCJD_2_ isolate after 1 passage in TgMet/Val_129_ mice and subsequent inoculation into TgVal_129_ mice (Table 3). These observations support the hypothesis that adaptation of BSE agent to the human-PrP Met_129_ amino-acid sequence promotes its transmission to human PrP Val_129_–expressing hosts.

### vCJD Prions in TgVal_129_ Mice 

Challenge of TgMet_129_ or TgMet/Val_129_ mice with vCJD prions resulted in faithful propagation of a typical PrP^vCJD^ (also named type 4), characterized by low size fragments (19-kDa fragment for the aglycosyl band) and prominent diglycosylated species on WB (Figure 1, panel A, lanes 2, 3). These biochemical properties were accompanied by the key neuropathological hallmark of vCJD, the presence of abundant florid PrP plaques determined by immunohistochemical analysis of the brain (*31*) (data not shown).

In contrast, TgMet_129_–passaged vCJD-inoculated TgVal_129_ mice propagated a PrP^Sc^ with a WB signature that shared the same predominance of the diglycosylated glycoform seen in type 4 PrP^Sc^ but was distinguished by PK digestion products of greater molecular mass (Figure 1, panel A, lanes 4, 6), which closely resemble those seen in human type 2 PrP^Sc^ (Figure 1, panel A, lane 8). This differential biochemical pattern is associated with the presence of amyloid plaques restricted to the corpus callosum without a florid morphology. Moreover, we saw no specific vacuolar changes in the brains of these animals. PET blot analysis of these brains confirmed PrP^Sc^ deposition in corpus callosum and head of caudate nucleus in the brain of vCJD-inoculated TgVal_129_ mice (Figure 2, panels D, E). However, PrP^Sc^ deposition was quite limited in comparison with those observed in vCJD-inoculated TgMet_129_ (Figure 2, panels A, B) and TgMet/Val_129_ mice (Figure 2, panel C).

**Figure 3:**
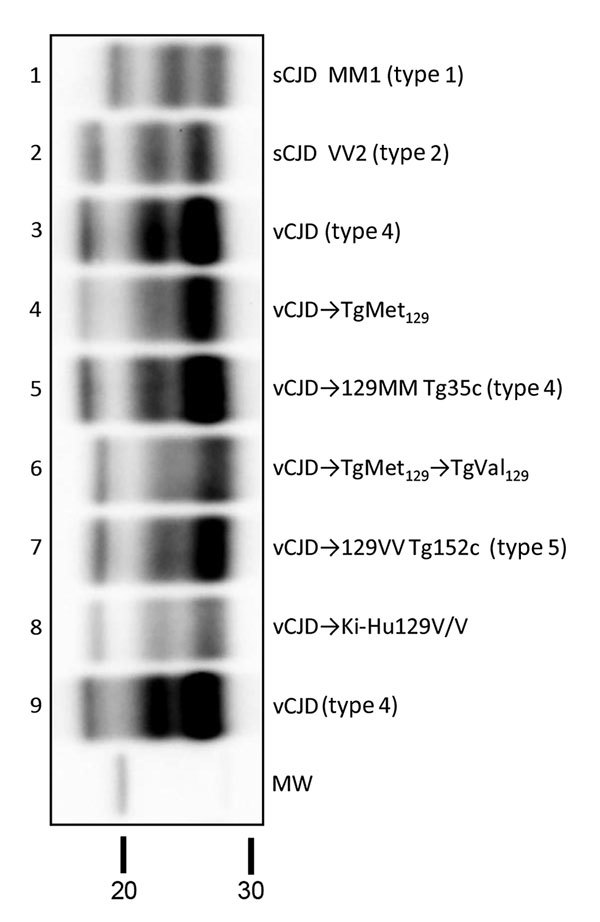
Biochemical comparison of brain protease-resistant prion protein (PrPres) detected in transgenic mice expressing prion protein Met129, and Val129 mice and inoculated with vCJD brain homogenate. Similar quantities of PrPres were loaded for adequate comparison, and immunoblots were detected by using Sha31 monoclonal antibody. Lanes 4 and 6 show passages from this study; lane 5 shows sample codification I-10629 and lane 7 sample codification I-11724 from the MRC Prion Unit in the United Kingdom (27); lane 8 shows sample codification #139-A5603 from Tohoku University Graduate School of Medicine, Sendai, Japan (30). The original vCJD isolate (Hu-vCJD2) used for mouse inoculations in this study was also included on the blot (lanes 3 and 9); sCJD MM1 (lane 1) and VV2 (lane 2) isolates were included for biochemical comparative purposes. Molecular weight (MW) in kDa is shown. CJD, Creutzfeldt-Jakob disease; sCJD, sporadic CJD; vCJD, variant CJD.

These results resemble those previously described in a different TgVal_129_ mouse line in which neuropathological and molecular features similar to those observed in our TgVal_129_ were characterized (*2*,*27*,*28*). To prove the same PrP^res^ molecular profile identity between this previously characterized PrP^Sc^ (called type 5 PrP^Sc^, vCJD**→**129VV Tg152c) and our vCJD-TgVal_129_ PrP^Sc^, we performed a biochemical characterization by WB and found no molecular profile differences in PrP^res^ from the various mouse lines (Figure 3, lanes 6 and 7). These particular molecular mass and glycoform profile characteristics seem to be a hallmark of vCJD transmission to human-PrP Val_129_, since these features were also found in a different human-PrP Val_129_ transgenic mouse line challenged with vCJD (vCJD**→**Ki-Hu129V/V) (*26*) (Figure 3, lane 8). These results, suggesting vCJD prion infection can result in the generation of distinct molecular and neuropathological phenotypes dependent on human-PrP polymorphic residue 129, are in accordance with those reported previously (*2*,*28*,*46*).

## Discussion

We report a detailed comparison of the transmission properties of BSE and vCJD prions among humanized transgenic mice with different *PRNP* codon 129 genotypes. Because a high expression level of PrP in transgenic mice directly influences prion disease susceptibility and incubation time, these transgenic mice have an advantage over knock-in mice for evaluating these features in the different human PrP genotypes. In addition, the 3 mouse models used in our study have equivalent PrP expression levels, making them suitable for studying comparative susceptibilities across the different *PRNP* codon 129 genotypes.

In previous reports, we demonstrated that Met_129_ homozygous individuals might be susceptible to a sheep or goat BSE agent to a higher degree than to cattle BSE and that these agents might transmit with molecular and neuropathological properties indistinguishable from those of vCJD (*31*). In this study, we wanted to extend these results to the other human *PRNP* genotypes: Met/Val_129_ and Val/Val_129_. We gained a different perspective when several BSE isolates adapted to different species inoculated in TgVal_129_ mice showed an apparent lack of transmission. In addition, almost all inoculated TgMet/Val_129_ mice did not transmit BSE; this finding supports the interpretation by Wadsworth et al. that human PrP Val_129_ severely restricts propagation of the BSE prion strain (*27*). 

An unexpected result of this study was the finding that 1 BSE isolate from a goat (Ca-BSE/Go) was clinically transmitted to 1 of 10 TgMet/Val_129_ mice and subclinically transmitted to TgVal_129_ mice. This particular isolate is characterized by a high infectious titer (*35*) that could explain the potential for this inoculum to overcome the restriction on BSE prions to propagate in TgVal_129_ mice.

Although cattle BSE did not transmit to TgMet/Val_129_ mice directly, adaptation of the BSE agent to human PrP Met_129_ sequence and subsequent inoculation of the resultant vCJD prions to TgMet/Val_129_ mice produced a 100% attack rate. However, we did not detect clinical prion disease, supporting a slower rate of vCJD conversion compared with that among TgMet_129_ mice. This slow but potential conversion rate in TgMet/Val_129_ mice correlates well with the single vCJD case of a human carrying the PrP Met/Val_129_ genotype (*22*) and with the description of subclinical secondary transmissions through human vCJD–infected tissues (*4*–*7*,*47*).

TgVal_129_ mice challenged with Hu-vCJD_2_ did not produce detectable brain PrP^res^ and clinical signs, in spite of the overexpression of HuPrP-Val_129_ and the use of the more efficient intracerebral route of infection. However, subclinical infection in these TgVal_129_ mice was demonstrated in BoPrP-Tg110 mice. These data suggest that PrP Val_129_ could sustain a very slow and limited vCJD conversion rate that is consistent with the detection of PrP^res^ in tonsils and appendixes of asymptomatic PrP Val_129_ persons (*23*–*25*). Previous studies of other transgenic mice expressing PrP Val_129_ have also shown a low transmission efficiency of vCJD (*2*,*27*,*30*).

The fluctuating subclinical transmissibility of both vCJD inocula in TgVal_129_ mice (negative for Hu-vCJD_1_ and positive for Hu-vCJD_2_) might be caused by differences in prion titer between inocula. This assessment was strengthened after the transmission of both vCJDs to TgMet_129_ mice, in which a shorter incubation period was observed in animals inoculated with Hu-vCJD_2_. A certain variability in subclinical transmissibility and incubation time between different vCJD isolates is not uncommon, as has been previously reported (*2*,*27*,*30*), suggesting that a Val_129_ transmission barrier can only be overcome with highly infectious vCJD isolates.

The dramatic changes in the susceptibility of TgVal_129_ mice (Table 3) challenged with vCJD isolates first passaged in TgMet_129_ mice suggest an apparent increase in titer of both vCJD prion isolates; however, adaptation of the inocula to the new host mouse cannot be disregarded as being partly responsible for this increased susceptibility. We observed a 100% infection rate, but without clinical signs of prion disease. We observed similar transmission features when we passaged vCJD in TgMet/Val_129_ mice. In addition, the apparent PrPVal_129_ restricted propagation of cattle BSE and BSE from other species was completely abolished after its adaptation to human PrPMet_129_.

Although PrP overexpression and the inoculation route can affect transmission efficiency, our results and those previously reported in both overexpressing and knock-in transgenic mice (*2*,*27*,*30*) suggest that the Val_129_ PrP variant could sustain a very slow and limited vCJD conversion rate, and is unable to completely prevent vCJD transmission. Biochemical and neuropathological features of vCJD transmission to TgVal_129_ mice showed substantial differences compared to TgMet_129_ or TgMet/Val_129_ mice. Similar to previous reports (*2*,*27*,*28*,*48*), a type 5 PrP^Sc^ associated with very weak and diffuse PrP plaques without a florid morphology was the hallmark among these mice. In addition, our demonstration of previously unreported type 5 PrP^Sc^ in brain samples of vCJD-challenged knock-in Ki-Hu129V/V mice (*30*) establishes that the evolution of type 5 PrP^Sc^ associated with the transmission of vCJD prions to the Val_129_ genotype is not an artifact of PrP overexpression. This finding further reinforces the specific biochemical features of vCJD when transmitted to the human-PrP Val_129_ sequence.

Extrapolation of results from prion transmission studies based on transgenic mice has to be done with caution, especially when human susceptibility to prions is analyzed. However, our results clearly indicate that PrPVal_129_ individuals are highly resistant to transmission of cattle BSE or BSE passaged in other species. Also, PrPVal_129_ individuals might be susceptible to infection with human-passaged BSE (vCJD) prions, and the propagated agents might transmit with molecular and neuropathological properties distinguishable from those of type 4 PrP^res^. Although the resultant type 5 PrP^Sc^ shares the same fragment sizes as those of type 2 PrP^Sc^, the 2 PrP^Sc^ types can be distinguished by the predominance of the diglycosylated glycoform associated with type 5 PrP^Sc^. Overall, our results indicate that human Val_129_-PrP polymorphic variant is a strong molecular protector against BSE zoonotic transmission but fails to prevent human-to-human vCJD transmission. Because potential late-onset vCJD cases could appear in the population (*49*,*50*) these findings underline the need for continued investigation of all forms of human prion disease.

Technical Appendix Transmission of Ca-BSE bovine spongiform encephalopathy–derived isolates adapted in different human prion protein polymorphic variants to BoPrP-Tg110 mice and **t**ransmission of variant Creutzfeldt-Jakob disease and cattle, sheep, and goat BSE isolates to TgVal129 (8×) mice.
