# Supplementary material for: Protective Effect of Val129-PrP against Bovine Spongiform Encephalopathy but not Variant Creutzfeldt-Jakob Disease
Source: Emerg Infect Dis. 2017 Sep;23(9):1522–30. doi: 10.3201/eid2309.161948 (PMC5572891; doi:10.3201/eid2309.161948)
Supplement: Technical Appendix — Transmission of Ca-BSE bovine spongiform encephalopathy–derived isolates adapted in different human prion protein polymorphic variants to BoPrP-Tg110 mice and transmission of variant Creutzfeldt-Jakob disease and cattle, sheep, and goat BSE isolates to TgVal129 (8×) mice. [file 16-1948-Techapp-s1.pdf]

Article DOI: https://doi.org/10.3201/eid2309.161948

Protective Effect of Val$_{129}$-PrP against Bovine Spongiform Encephalopathy but not Variant Creutzfeldt-Jakob Disease

Technical Appendix

Technical Appendix Table 1. Transmission of Ca-BSE derived isolates adapted in different human PrP polymorphic variants to BoPrP-Tg110 mice

	Mean survival time in days±SD (n/n$_0$)*†
Isolates	BoPrP-Tg110
Ca-BSE$_0$	303 ± 10 (13/13)(*1*)
Ca-BSE$_0$$\rightarrow$TgVal$_{129}$	>700‡ (0/7)
Ca-BSE$_2$	308 ± 5 (5/5) (*1*)
Ca-BSE$_2$$\rightarrow$TgMet$_{129}$$\rightarrow$TgMet$_{129}$	308 ± 13 (5/5)(*2*)
Ca-BSE/Sh(ARQ)	234 ± 5 (16/16)(*1*)
Ca-BSE/Sh(ARQ) $\rightarrow$TgVal$_{129}$	>700‡ (0/6)
Ca-BSE/Sh(ARQ) $\rightarrow$TgVal$_{129}$$\rightarrow$TgVal$_{129}$	>700‡ (0/5)
Ca-BSE/Sh(ARQ) $\rightarrow$TgMet$_{129}$	278 ± 7 (7/7)
Ca-BSE/Sh(ARQ) $\rightarrow$TgMet$_{129}$$\rightarrow$TgVal$_{129}$	311 ± 9 (6/6)
Ca-BSE/Go	239 ± 66 (6/6)(*3*)
Ca-BSE/Go$\rightarrow$TgVal$_{129}$	427 ± 38 (3/6)
Hu-vCJD$_1$	370 ± 33 (9/9)(*1*)
Hu-vCJD$_1$$\rightarrow$TgVal$_{129}$	>700[b] (0/5)
Hu-vCJD$_2$	276 ± 9 (6/6)
Hu-vCJD$_2$$\rightarrow$TgVal$_{129}$	371 ± 5 (3/3)§
Hu-vCJD$_2$$\rightarrow$TgMet/Val$_{129}$$\rightarrow$TgVal$_{129}$	260 ± 41 (6/6)

*n/n$_0$: diseased PrP^{res} positive/inoculated animals.
†Survival time is indicated as mean±SD dpi of all the mice scored positive for PrP^{res}.
‡Animals were euthanized without clinical signs at 700 dpi.
§Three additional animals had to be culled before the end of the experiment because of intercurrent illnesses; all were negative for brain PrP^{res} on WB.

Technical Appendix Table 2. Transmission of vCJD and cattle, sheep and goat BSE isolates to TgVal$_{129}$ (8x) mice

	Mean survival time in days ±SD (n/n$_0$)*	
	TgVal$_{129}$ (8x)	
Isolates	1st passage	2nd passage
Hu-sCJD MM1	295 ± 21 (6/6)	215 ± 4 (7/7)
Hu-sCJD VV2	228 ± 7 (5/5)	228 ± 14 (5/5)
Hu-TSE negative	>700† (0/6)	>700† (0/6)
Ca-BSE$_0$	>700† (0/6)	>700† (0/5)
Ca-BSE$_2$	>700† (0/3)‡	>700† (0/3)‡
Ca-BSE/Sh(ARQ)	>700† (0/5)	>700† (0/6)
Ca-BSE/Go	>700† (0/5)	
Hu-vCJD$_2$	>700† (0/5)	>700† (0/6)

*Survival time is indicated as mean±SD dpi of all the mice scored positive for PrP^{res}. n/n$_0$: diseased PrP^{res} positive/inoculated animals.
†Animals were euthanized without clinical signs at 700 dpi.
‡Three additional animals had to be culled before the end of the experiment because of intercurrent diseases; all were negative for brain PrP^{res} on WB.

References

1. Padilla D, Béringue V, Espinosa JC, Andreoletti O, Jaumain E, Reine F, et al. Sheep and goat BSE propagate more efficiently than cattle BSE in human PrP transgenic mice. PLoS Pathog. 2011;7:e1001319. PubMed http://dx.doi.org/10.1371/journal.ppat.1001319

2. Torres JM, Espinosa JC, Aguilar-Calvo P, Herva ME, Relaño-Ginés A, Villa-Diaz A, et al. Elements modulating the prion species barrier and its passage consequences. PLoS One. 2014;9:e89722. PubMed http://dx.doi.org/10.1371/journal.pone.0089722
3. Aguilar-Calvo P, Fast C, Tauscher K, Espinosa JC, Groschup MH, Nadeem M, et al. Effect of Q211 and K222 PRNP polymorphic variants in the susceptibility of goats to oral infection with goat bovine spongiform encephalopathy. J Infect Dis. 2015;212:664–72. PubMed http://dx.doi.org/10.1093/infdis/jiv112
